# Dopamine-Induced Conformational Changes in Alpha-Synuclein

**DOI:** 10.1371/journal.pone.0006906

**Published:** 2009-09-04

**Authors:** Tiago F. Outeiro, Jochen Klucken, Kathryn Bercury, Julie Tetzlaff, Preeti Putcha, Luis M. A. Oliveira, Alexandre Quintas, Pamela J. McLean, Bradley T. Hyman

**Affiliations:** 1 MassGeneral Institute for Neurodegenerative Disease, Alzheimer Research Unit, Massachusetts General Hospital, Charlestown, Massachusetts, United States of America; 2 Harvard Medical School, Boston, Massachusetts, United States of America; 3 Cell and Molecular Neuroscience Unit, Instituto de Medicina Molecular, and Instituto de Fisiologia, Faculdade de Medicina da Universidade de Lisboa, Lisboa, Portugal; 4 Division of Molecular Neurology, University Hospital Erlangen, Erlangen, Germany; 5 Laboratório de Patologia Molecular, Instituto Superior de Ciências da Saúde Egas Moniz, Monte da Caparica, Portugal; National Institutes of Health, United States of America

## Abstract

**Background:**

Oligomerization and aggregation of α-synuclein molecules play a major role in neuronal dysfunction and loss in Parkinson's disease [1]. However, α-synuclein oligomerization and aggregation have mostly been detected indirectly in cells using detergent extraction methods [2], [3], [4]. A number of in vitro studies showed that dopamine can modulate the aggregation of α-synuclein by inhibiting the formation of or by disaggregating amyloid fibrils [5], [6], [7].

**Methodology/Principal Findings:**

Here, we show that α-synuclein adopts a variety of conformations in primary neuronal cultures using fluorescence lifetime imaging microscopy (FLIM). Importantly, we found that dopamine, but not dopamine agonists, induced conformational changes in α-synuclein which could be prevented by blocking dopamine transport into the cell. Dopamine also induced conformational changes in α-synuclein expressed in neuronal cell lines, and these changes were also associated with alterations in oligomeric/aggregated species.

**Conclusion/Significance:**

Our results show, for the first time, a direct effect of dopamine on the conformation of α-synuclein in neurons, which may help explain the increased vulnerability of dopaminergic neurons in Parkinson's disease.

## Introduction

Protein misfolding and aggregation, processes involved in several neurodegenerative diseases, are likely preceded by conformational changes in the proteins involved [8]. The transient nature and the small scale of these conformational changes have made them extremely difficult to study directly.

Recent studies have shown that natively unfolded molecules can partially fold and form, in vitro, either toxic oligomeric species or microscopic fibrillar aggregates, which are neurotoxic. How, why, and when misfolding happens in vivo is still unclear [1].

α-Synuclein (aSyn), a small (140 amino acid) neuronal protein of unknown function which is ubiquitously expressed in the brain, displays little secondary structure in vitro and belongs to a group of proteins known as ‘natively unfolded’ [9], [10]. Under certain conditions, aSyn can adopt specific conformations in association with model lipids or in the presence of detergents [11], [12], [13].

In PD, there is substantial loss of dopaminergic neurons in the substantia nigra, with the presence of fibrillar inclusions called Lewy bodies (LBs) comprising aSyn as a major constituent [14]. Diseases associated with the accumulation of fibrillar forms of aSyn are commonly known as synucleinopathies. The preferential vulnerability of dopaminergic neurons in PD is unclear, but a link between dopamine biology and aSyn as been hypothesized [15], since dopamine was shown to form adducts with aSyn in the test tube, appears to stabilize protofibrillar forms of aSyn, and inhibits aSyn fibril formation in vitro [5]. Recently, dopamine-modified aSyn was shown to block chaperone mediated autophagy [16], but the full spectrum of effects of this dopamine interaction with aSyn in living cells is still obscure. One possibility is that this is part of the normal function of aSyn, but it could also bear a connection with the increased vulnerability of dopaminergic neurons.

To explore this question further, we developed a method that specifically detects aSyn conformational alterations within cells, using a highly sensitive and specific assay of molecular proximity called fluorescence lifetime imaging microscopy (FLIM). Here, we applied FLIM to investigate the effect of dopamine and other chemical modulators of neuronal activity on the conformation of aSyn in primary neurons. A deeper understanding of the connection between aSyn and dopamine has implications for current and future PD therapeutic interventions.

## Materials and Methods

### Plasmid construction

The constructs for human wild type untagged aSyn have been described previously [17], [18], [19], [20], [21]. Briefly, cDNA encoding the genes were cloned into pcDNA3.1 (Invitrogen, CA, USA) expression vectors. N- and/or C-terminal Myc or V5 tags were generated using annealed oligomers coding for Myc or V5, and subcloned into wild type aSyn expressing pcDNA3.1 plasmid.

### Cell culture, transfection, and immunocytochemistry

Human H4 neuroglioma cells (HTB-148 - ATCC, Manassas, VA, USA) were maintained in OPTI-MEM (Invitrogen, CA, USA) supplemented with 10% fetal bovine serum. H4 cells were passaged 24 hours prior to transfection and plated in four-well chamber slides for immunocytochemistry (Labtek, Nalgen-Nunc, Naperville, IL, USA). Cells were transfected with equimolar ratios of plasmids using Superfect (Qiagen, Chatsworth, CA, USA) according to the manufacturer's instructions. After 24 hours cells were washed with phosphate buffered saline (PBS), and fixed with 4% paraformaldehyde for 10 min at room temperature (RT). After washing with PBS cells were permeabilized in tris buffered saline (TBS) containing 0.1% Triton X-100 for 20 min at RT. After blocking in 1.5% normal goat serum containing TBS for 1 hour cells were incubated with primary antibody for 2 hours at RT or overnight at 4°C (mouse anti-Myc 1∶1000, Abcam, Cambridge, MA, USA; rabbit anti-V5 1∶3000, AB9116, Abcam, Cambridge, MA, USA) followed by washing with PBS and secondary antibody incubation for 1 hour (goat anti-rat IgG-Alexa488, 1∶300, Molecular Probes, Eugene, OR, USA; goat anti-rabbit IgG-Cy3 1∶500, Jackson Immunoresearch, PA, USA). After a final wash, slides were mounted with aqueous mounting solution (GVA, Zymed, San Francisco, CA, USA) and subjected to fluorescence microscopy and fluorescence lifetime imaging microscopy (FLIM).

HEK cells were maintained in DMEM with 10% FBS and handled as described above for the H4 cells. MES23.5 cells, received with permission from Dr. Stanley Appel were maintained as described [22] and transfected using Lipofectamine 2000 according to the manufacturer's instructions.

### SDS-PAGE and immunoblotting

24 hours after transfection, H4 cells were washed with cold PBS, harvested by scraping in cold lysis buffer without detergents (Tris/HCl 50 mM pH 7.4, NaCl 175 mM, EDTA 5 mM pH 8.0, protease inhibitor cocktail, Roche, Basel, CH) and sonicated for 10 seconds. Lysates were cleared from debris by a 9,500 g centrifugation for 10 min at 4°C and were then subjected to SDS-PAGE using 10–20% gradient Tris–Glycine gels (Novex, San Diego, CA, USA) for Western blot analysis. Protein bands on the SDS-PAGE were transferred to Immobilon-P membrane (Millipore, Bedford, MA, USA) and blocked in blocking buffer (Lycor, Lincoln, NE, USA) for 1 hour prior to the addition of the primary antibody at room temperature for 1–2 hours or overnight at 4°C. The blots were washed three times in TBS with 0.2% Tween (TBS-T, pH 7.4), and were incubated at room temperature for 1 hour in fluorescent labeled secondary antibodies (IRDye 800 anti-rabbit or anti-mouse, Rockland Immunochemicals, Gilbertsville, PA, USA 1∶3000 or Alexa-680 anti-rabbit or anti-mouse, Molecular Probes, Eugene, OR, USA 1∶3000). After washing three times in TBS-T immunoblots were analyzed and quantified using the Odyssey infrared imaging system (Lycor, Lincoln, NE, USA).

### Fluorescence lifetime imaging microscopy and calculation

FLIM has been recently described as a technique for the analysis of protein proximity [23], [24], [25]. The technique is based on the observation that fluorescence lifetimes of a donor fluorophore shorten if it is in close proximity (<10 nm) to a FRET acceptor. The decrease in lifetime is proportional to the distance between the fluorophores at R^6^. A mode-locked Ti-sapphire laser (Spectra-Physics, Fremont, California) emits a femtosecond pulse every 12 nanoseconds to excite the fluorophore. A high-speed Hamamatsu (Bridgewater, New Jersey) detector and hardware/software (SPC-830 Becker and Hickl, Berlin, Germany) were used to measure fluorescence lifetimes on a pixel-by-pixel basis. Donor fluorophore (Alexa488) lifetimes were fit to two-exponential decay. One component was fixed to the expected lifetime of Alexa488 without an acceptor (Cy3) in close proximity for energy transfer (negative control – monofit) that was determined by fitting to one-exponential decay curve (mean lifetime monofit). To validate the two component fit procedure, the same cells from the negative control mono-fit were subjected to two exponential decay curve fitting and revealed the experimental value for Alexa488 lifetime that did not differ from the mono-fit lifetime and was used in the experiment as calculated negative control. As a positive control, Alexa 488 lifetime was measured in the presence of a FRET acceptor (Cy3) in close proximity presenting the acceptor with a donkey anti-goat Cy3 labeled Ab, directed against the goat anti-mouse Alexa 488 secondary Ab used to visualize the anti-V5 monoclonal antibody [26]. All combinations of Myc-, V5-, or un-tagged aSyn molecules were stained using the same antibody combination as described above. As a positive control, Alexa488 lifetime was measured in the presence of a FRET acceptor (Cy3) in close proximity [23] presenting the acceptor with a donkey anti-goat Cy3 labeled antibody, directed against the goat anti-mouse Alexa488 secondary antibody used to visualize the anti-V5 monoclonal antibody. Experiments were performed in triplicate using the number of cells indicated. Results are expressed as mean fluorescence lifetime ± SEM.

### Detergent-Solubility Franctionation and Gel Electrophoresis

Detergent solubility was performed by adding Triton X-100 to total cell lysates (final concentration 1%) and incubating for 30 min on ice followed by centrifugation (15,000×*g*, 60 min, 4°C). The supernatant was designated Triton X-100 soluble fraction, and the pellet was redissolved in 2% SDS-containing lysis buffer and sonicated for 10 s (Triton X-100 insoluble fraction). Additional washing of the Triton X-100 insoluble pellet was found to not alter the aSyn content in this fraction (data not shown) and was omitted from the experiments. Protein concentration was determined using a BCA (Pierce, IL, USA) protein assay. 20–40 µg of each cell lysate was loaded onto 4–20 or 10–20% gradient Tris/glycine gels (Invitrogen, CA, USA) for Western blot analysis. SDS-PAGE was performed with SDS containing commercially available standard running and sample loading buffers (Invitrogen, CA, USA). Protein was transferred to Immobilon-P membrane (Millipore, Bedford, MA) and blocked in blocking buffer (Lycor, Lincoln, NE) for 1 hour prior to the addition of primary antibody, anti-aSyn (Syn-1, 1∶1000, BD Transduction Laboratories) at room temperature for 1–2 hours or overnight at 4°C. Following three Tris-buffered saline with Tween 20 washes, infrared fluorescent-labeled secondary antibodies (IRDye 800 anti-rabbit or anti-mouse, Rockland Immunochemicals, Gilbertsville, PA, at 1∶3000 or Alexa-680 anti-rabbit or anti-mouse, Molecular Probes, Eugene, OR at 1∶3000) were incubated at room temperature for 1 hour and immunoblots were processed and quantified using the Odyssey infrared-imaging system (Lycor). Blots were also probed for actinin (anti-actinin, Sigma).

### Primary Cortical Neuronal Cultures

CD-1 mice and Sasco Sprague Dawley rats were obtained from Charles River laboratories. A cesarean section was performed and E14 mice or E18 rats were removed. The animals were decapitated and their cortices were removed and placed in Phosphate Buffered Saline (PBS). The tissue was manually triturated in 10% Fetal Bovine Serum (FBS) from, and Neurobasal medium (Invitrogen, CA, USA) and plated onto 4 well chamber slides (Lab tek) or 100 mm tissue culture dishes (Corning). The slides or dishes were coated with Poly-D-Lysine (Sigma) 24 hours prior to the dissection and incubated with Human Placenta laminin (Sigma) and Penicillin Streptomycin (Invitrogen, CA, USA) in Neurobasal Medium overnight at 37°C. Cells were plated in 10% fetal bovine serum (Invitrogen, CA, USA) in Neurobasal medium. One hour later, the media was removed and replaced with B-27 and Neurobasal medium. The cells were maintained and fed in the same media every 5–7 days depending on their density.

### Transfection of Primary Cortical Neurons

Cortical neurons were plated on 4 well glass bottom chamber slides (Nunc). Cells were maintained in Neurobasal media (Invitrogen, CA, USA) containing B-27 (Invitrogen, CA, USA) and penicillin streptomycin (Invitrogen, CA, USA). Between 5 and 7 days in vitro (DIV) cells were transiently transfected using Lipofectamine 2000 (Invitrogen). A concentration of 2 µg DNA/ 5 µl of Lipofectamine 2000 was used per each well of the chamber slide. The DNA and Lipofectamine 2000 were added into DMEM (Invitrogen, CA, USA) and incubated separately for 5–15 minutes before being combined. The DNA complex was gently mixed and incubated for 45 minutes at room temperature. The neuronal maintenance media was removed from the cells and they were washed with phosphate buffered saline (PBS) containing no calcium or magnesium. The DNA complex was added to the cells for 2–6 hours at 37°C. The DNA complex was then removed and replaced with neuronal maintenance media. Cells were then fixed and processed for immunocytochemistry.

### Expression and Purification of human wt aSyn

The expression and purification procedure of human WT aSyn was a modified version of a previously described method [27]. Briefly, cells of *E. coli* strain BL-21 (GE Healthcare, NJ, USA) were transformed with the appropriate expression vector, and expression was induced by the addition of isopropyl _D_-thiogalactopyranoside at a final concentration of 1 mM. Cells were harvested, resuspended in 50 mM Tris (pH 8.5), 50 mM KCl, 5 mM MgAc, 0.1% NaN_3_ and 300 µM PMSF, and lysed by three passages through a French cell press. The extract was centrifuged at 18000 g at 4°C for 30 min to eliminate cell debris. The supernatant was saved and boiled for 20 min. The boiled extract was centrifuged at 45000 g at 4°C for 45 min and the supernatant was filtered with a 0.2 µm filter to remove possible pellet contamination. The aSyn containing extract was loaded on to an ion-exchange chromatography Q Sepharose™ (GE Healthcare, NJ, USA) fast flow column equilibrated with 20 mM Tris/HCl (pH 8.0). Proteins were eluted with a linear NaCl gradient (0.12–0.5 M) at a flow rate of 1.5 ml.min^−1^ and the eluate was monitored at 280 nm. Protein-containing fractions were collected and probed by western blot analysis using Syn-1 anti-aSyn antibody (BD Transduction Laboratories, CA, USA). Fractions containing aSyn were collected, concentrated by centrifugation using Amicon filters (Millipore) and applied to a gel filtration Superdex 75 column (GE Healthcare, NJ, USA), equilibrated with 50 mM Tris/HCl buffer (pH 7.5) containing 150 mM NaCl. Proteins were eluted with the same buffer at a flow rate of 1 ml.min^−1^. Again, fractions containing aSyn, probed by western blot, were collected and combined for dialysis against water and then lyophilized for future analysis.

### In vitro modification of purified α-syn by dopamine

Purified native aSyn (70 µM) was incubated with dopamine (Sigma) at a final concentration of 1, 10, 100 and 1000 µM in 50 mM sodium phosphate buffer (pH 7.4) at 37°C for 1 week in sterile conditions. aSyn concentration was determined spectrophotometrically (ε_275_ = 5974 M^−1^.cm^−1^) in a UV-Visible Jasco V-530 spectrometer.

### Far-UV circular dichroism (CD) spectroscopy

Secondary structure analysis was performed by far-UV (185–260 nm) CD in a Jasco J810 spectropolarimeter at 37°C (Julabo F25 temperature control unit) with a 0.01 cm path length. CD spectra were deconvoluted using CDSSTR algorithm [28] on Dichroweb (http://dichroweb.cryst.bbk.ac.uk/html/home.shtml) [29], [30]. All spectra were solvent baseline-corrected.

## Results

**Figure 1:**
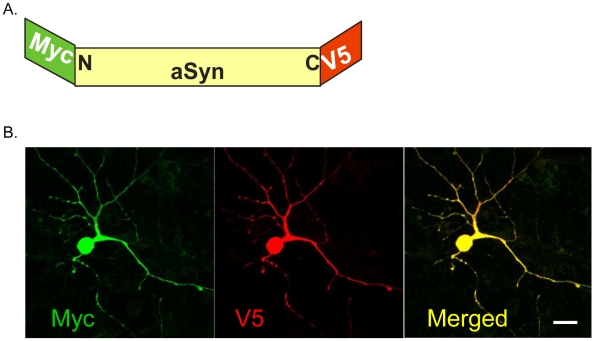
Expression of aSyn in primary cortical neurons. A. Schematic of the Myc-aSyn-V5 construct. B. Immunocytochemistry with anti-Myc (green) and anti-V5 (red) antibodies showing expression throughout the cell (including in the nucleus).

We have previously shown that conformational changes in aSyn can be monitored in immortalized H4 cells using the sensitive fluorescence resonance energy transfer (FRET) based proximity assay, FLIM [31]. To directly study the interaction between the amino-terminus and the carboxyl terminus of aSyn in neurons we overexpressed doubly-tagged Myc-aSyn-V5 in both mouse and rat primary neuronal cultures. Neurons were transfected at 5–7 days in vitro (DIV) using Lipofectamine 2000 and transfection efficiencies in the order of 5% were achieved. Immunostaining using antibodies against Myc and V5 confirmed that both epitope tags were expressed and completely colocalized at the subcellular level (Fig. 1A, B).

**Figure 2:**
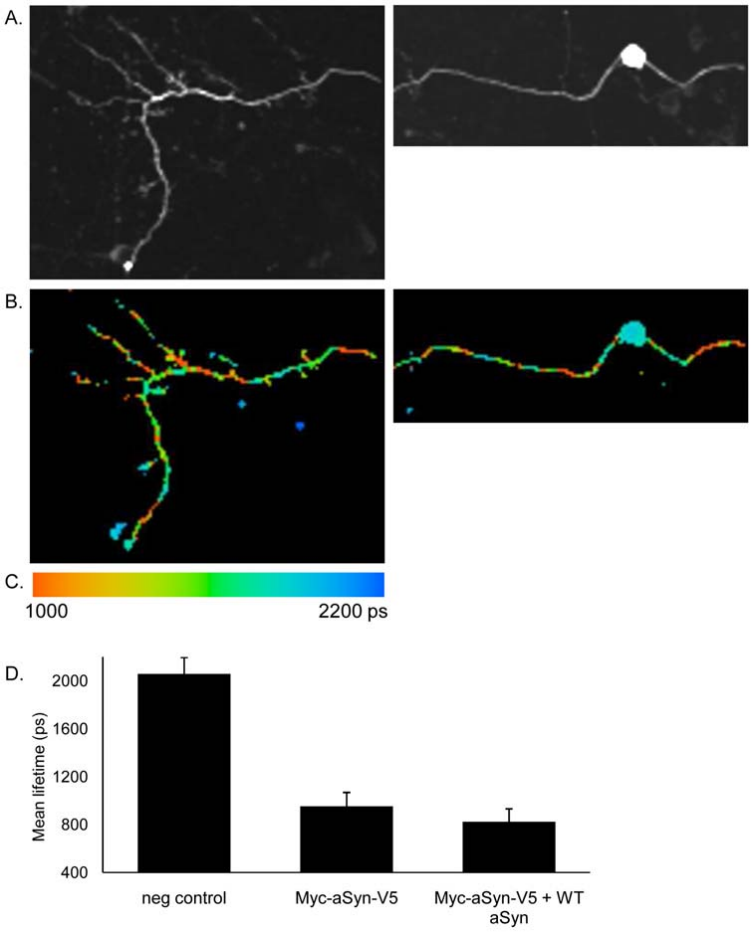
aSyn adopts different conformations in primary neuronal cultures. Primary cortical neurons were transfected with Myc-aSyn-V5 and immunocytochemistry was performed as in 1. A. Intensity image. B. Representative FLIM image showing the conformation of aSyn varies throughout the processes. C. Lifetime scale. D. Analysis of the contribution of intermolecular interactions for the lifetimes registered for aSyn. Dilution of the intermolecular interactions with untagged WT aSyn shows the majority of the detected interactions result from intramolecular interactions, i.e., different conformational states of aSyn (n = 3, 30–40 cells per condition).

Next, we used FLIM to examine the association between the N- and C-termini of aSyn in neurons. The N-terminus was labeled with the donor fluorophore, Alexa488, and the C-terminus was labeled with the acceptor molecule, Cy3 (Fig. 2A). When we examined the lifetime of the donor fluorophore we detected a striking range of lifetimes throughout the transfected neurons with significantly different lifetime being detected in the nucleus/cell body and throughout the neurites, as demonstrated by the differences in color coding throughout the neurons. These data indicate that aSyn adopts different conformations in specific subcellular environments (Fig. 2B, C). Interestingly, the donor fluorophore lifetime was consistently longer in the cell body/nucleus (∼1900 psec) than in the processes (∼1000 psec) suggesting that aSyn adopts a folded conformation in the processes because shortening of the lifetime corresponds to the fluorophores being in closer proximity to one another. Control experiments where the C-terminus of aSyn (V5) was labeled with the donor fluorophore, Alexa488, and the N-terminus (Myc) was labeled with Cy3, yielded similar results (data not shown) and all subsequent experiments were performed using the conditions described above.

In order to assess whether the different lifetimes were indicative of conformational changes (intramolecular interactions) or indicative of interactions between distinct aSyn molecules (intermolecular interactions), we co-transfected neurons with the epitope tagged aSyn along with untagged WT aSyn. In this situation, we observed that the lifetimes did not change, when compared to those observed for the tagged aSyn, indicating the majority of the interactions we detected were intramolecular, i.e., due to conformational changes in aSyn (Fig. 2D).

**Figure 3:**
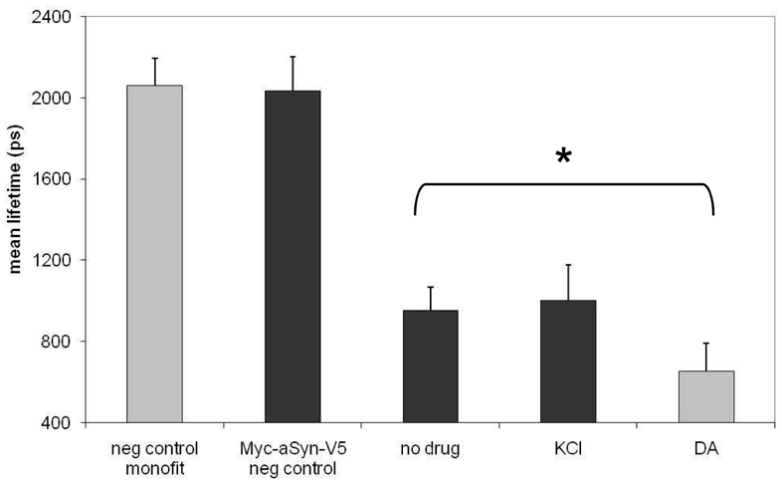
FLIM analysis of aSyn conformation in the presence of modulators of neuronal activity (KCl and Dopamine). KCl does not alter aSyn conformation (lifetime ∼1000 ps) whereas dopamine induces statistically significant changes (lifetime ∼650 ps) (*n = 3, ∼50 cells for each condition, p<0.01; unpaired, double sided t test).

**Figure 4:**
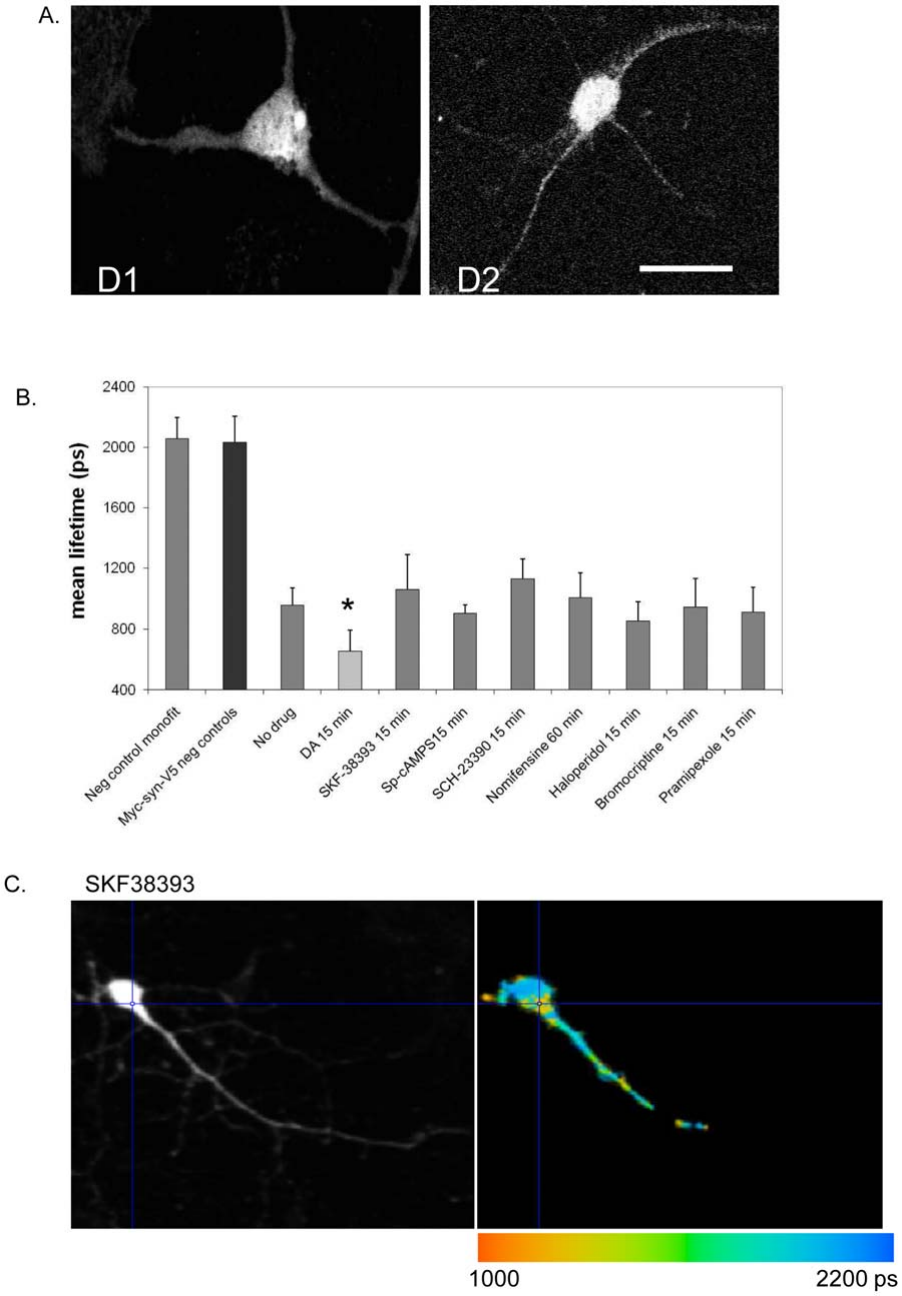
Dopamine receptor agonists and antagonists do not alter aSyn conformation. A. D1 and D2 receptors are present in primary cortical neurons. DIV7 neurons were immunostained against D1 and D2 receptors and observed via fluorescence microscopy. B. FLIM study with dopamine agonists/antagonists showing that these drugs do not alter aSyn conformation (*n = 3, 25–30 cells per condition, p<0.01; unpaired, double sided t test). C. FLIM image of an example compound, SKF38393, showing aSyn conformation is not altered.

Given the data suggesting that, in vitro, dopamine can impact aSyn conformation [5], [6], [7] we next asked if exposing aSyn transfected neurons to dopamine would affect aSyn conformation. Primary neurons overexpressing aSyn were treated with 100 µM dopamine (DA) for 10 min. DA significantly decreased the lifetime of the donor fluorophore to ∼650 ps (n = 3 independent experiments, total of 57 cells, p<0.01) at the dose tested, indicating that DA induces the N- and C-termini of aSyn to be in closer proximity, reflecting a change in conformation (Fig. 3). By contrast, various other treatments, including treating cells with 60 mM KCl for 10 minutes led to no changes in donor lifetime (Fig. 3). To tease apart the molecular mechanisms of the observed DA effect we examined the effect of DA agonists and antagonists on aSyn conformation. Primary neuronal cultures were immunostained using antibodies against D1 and D2 receptors to verify the presence of these receptors in our cultured neurons (Fig. 4A). We then screened a panel of well-characterized DA agonists and antagonists to determine if the previously observed conformational changes in aSyn could be mimicked or impeded (Table 1). Surprisingly, we were unable to detect an effect on aSyn conformation with any of the DA agonists or antagonists (Fig. 4B–C).

**Table 1:** Compounds tested in the FLIM assay in primary neurons.

Compound	Activity	Fluorescence lifetime (ps)	Effect on aSyn conformation
SKF-38393	Agonist	1058±232	−
Sp-cAMPS	Agonist	900±56	−
Quinpirole	Agonist	938±96	−
Ropinirole	Agonist	1014±108	−
Dopamine	−	632±140*	+
SCH-23390	Antagonist	1131±130	−
Haloperidol	Antagonist	852±123	−
Nomifensine	DAT blocker	1004±162	−
Dopamine + Nomifensine	−	959±89	−

Compounds with different activities were used and their effects on the conformation of aSyn were assessed via the FLIM assay. Only dopamine affected the conformation of aSyn (+) (*n = 3, 30–50 cells per condition, p<0.01).

**Figure 5:**
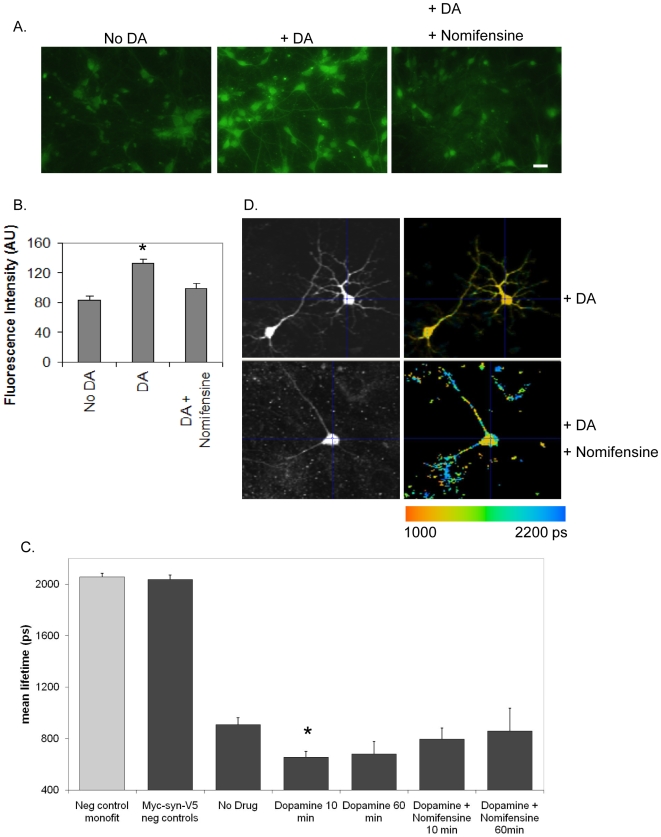
Dopamine enters cells to modulate aSyn conformation. A. ICC of cells treated with DA or DA + nomifensine (catecholamine uptake blocker) showing DA is able to enter cells. B. Quantification of the fluorescence intensity in A (∼200 cells) (*p<0.01, t test). C. FLIM analysis showing that DA needs to enter cells to alter aSyn conformation (n = 3, 25–30 cells per condition, *p<0.01; unpaired, double sided t test). D. Representative FLIM images of C.

To further determine if dopamine induced a general effect on the neurons or whether it was required to enter the cells in order to induce the conformational change in aSyn, we used nomifensine (100 µM) to block the activity of the dopamine transporter (Fig. 5A). Interestingly, we detected a reduction in the dopamine-induced conformational change in aSyn, suggesting that dopamine must gain entry into the cells in order to exert its activity on aSyn (Fig. 5A–D).

**Figure 6:**
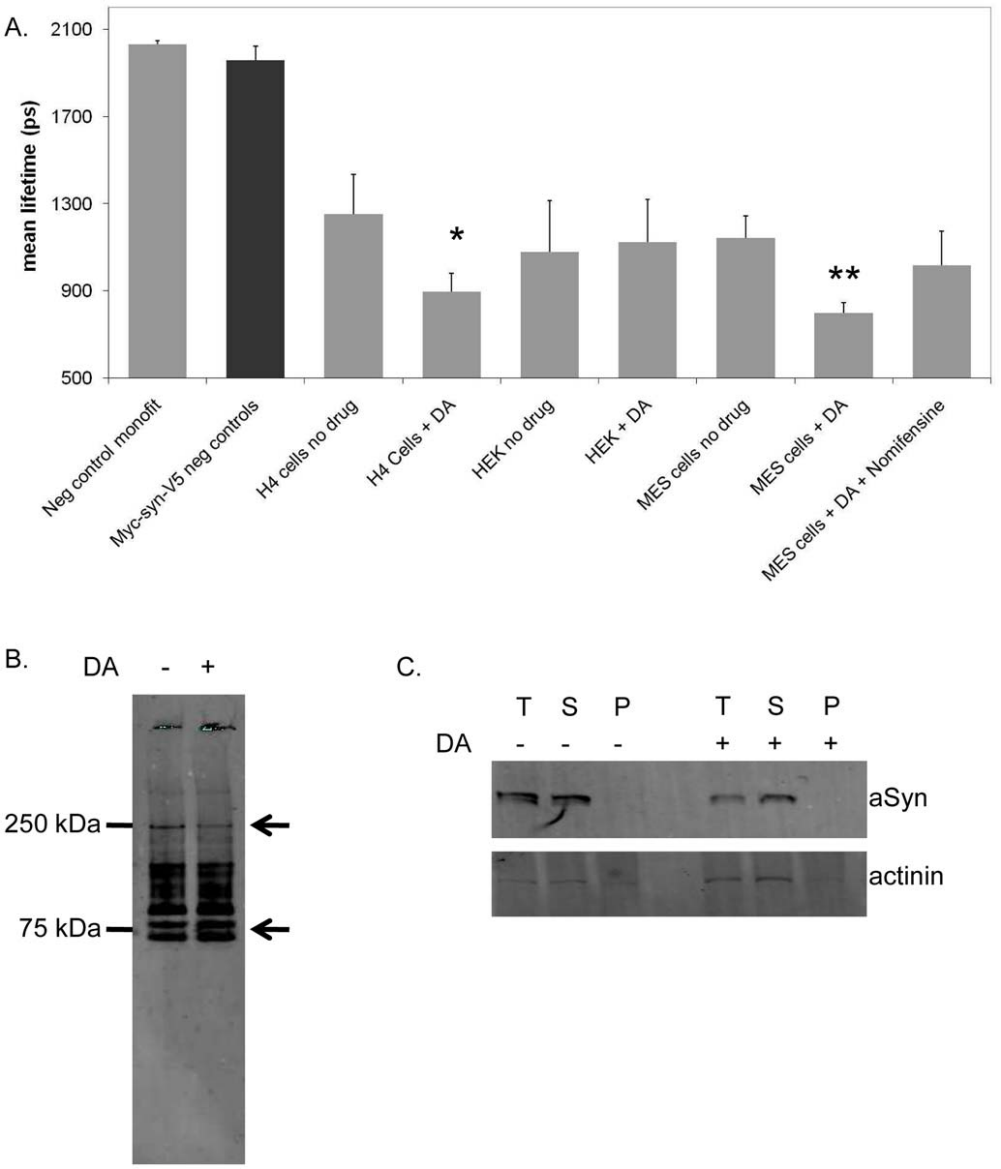
DA induces conformational changes in aSyn in neuronal cell lines. A. FLIM study showing DA alters aSyn conformation in cell lines of neuronal origin (H4 and MES23.5) but not in HEK cells (*n = 3, 30–40 cells per condition, p<0.01; unpaired, double sided t test). B. Native PAGE showing the presence of certain oligomeric species (arrows) is altered in DA treated H4 cells (representative immunoblot shown, n = 3). C.Triton X-100 fractionation showing DA does not alter the soluble vs. insoluble aSyn fraction in H4 cells (T-total, S-supernatant, P-pellet) (representative immunoblot shown, n = 3).

To further investigate how the conformational changes in aSyn affect its biochemical properties, we sought to recapitulate the modulation of conformation in immortalized cell lines, which would enable us to achieve higher transfection efficiencies required for biochemical studies. First, we transfected three cell lines of different origins (H4, MES23.5, and HEK) with the Myc-aSyn-V5 construct. These cells were then treated with dopamine and processed for FLIM. Interestingly, we found that the in the cell lines of neuronal origin (H4 and MES23.5) dopamine induced a conformational change similar to that observed in primary neuronal cultures (Fig. 6A). In limited experiments using HEK cells, we did not observe such a conformational change.

We previously identified, in brain tissue derived from patients with dementia with Lewy bodies (DLB), in aSyn transgenic mice, and in aSyn H4 expressing cells, oligomeric aSyn species which are detergent-insoluble [17]. Here, we hypothesized that the DA-induced conformational changes in aSyn might affect its detergent solubility. To investigate whether DA affected aSyn oligomerization we used native polyacrylamide gel electrophoresis (PAGE). In cells treated with DA, we observed a ∼50% decrease in a ∼250 KDa band and a ∼25% increase in two ∼75 KDa bands, demonstrating that DA induces slight but detectable changes in aSyn oligomerization (Fig. 6B, arrows). To further assess the effect of DA on aSyn solubility we performed a triton X-100 detergent fractionation of H4 cell lysates expressing Myc-aSyn-V5 treated or untreated with DA. We found no significant differences in the solubility of aSyn in either groups of cells (Fig. 6C).

**Figure 7:**
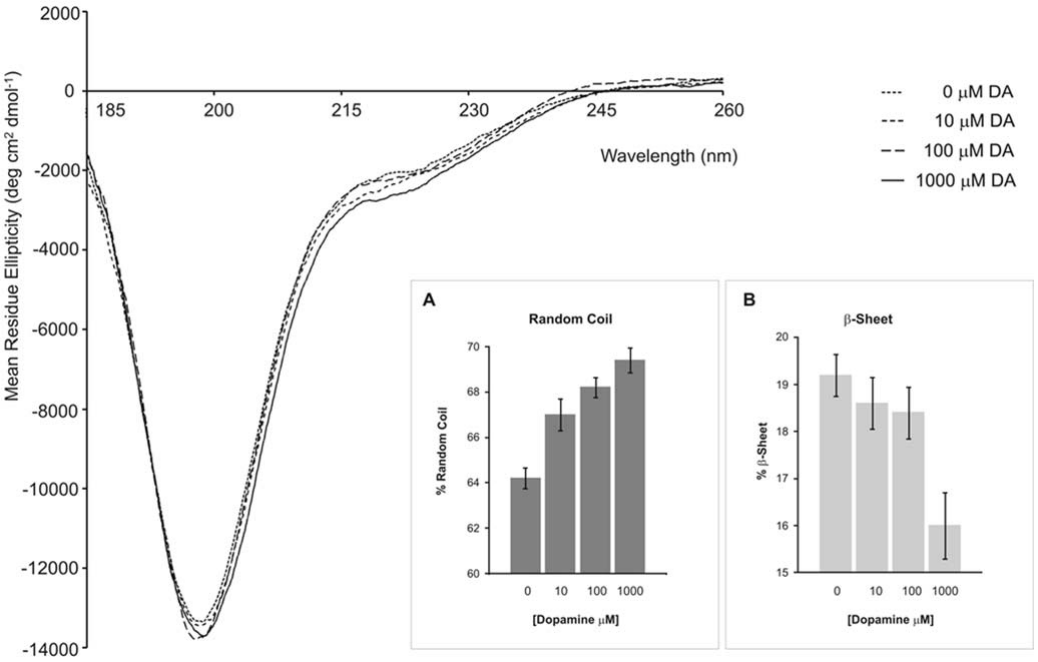
aSyn secondary structure alterations induced by DA. Conformational changes of aSyn in the absence (....) and in the presence of 10 µM (- - -); 100 µM (-- --) and 1000 µM (___) of dopamine were monitored by CD. The concentration of aSyn used was 70 µM and the spectra were taken at 37°C in a 0.1 mm path quartz cuvette at 25°C. The inset graphs show the percentage of random coil and beta-sheet as a function of DA concentration and was calculated after deconvolution of the CD spectra according to the description in the material and methods section (average of 5 independent experiments +/− SD).

In order to determine whether DA induces changes at the level of the secondary structure of aSyn we used circular dichroism (CD). Spectra were taken at 37°C in the absence or in the presence of different DA concentrations (10, 100 and 1000 µM of DA, corresponding to ratios of DA:aSyn of 0.14; 1.4 and 14.0) for a fixed concentration of aSyn of 70 µM (Fig. 7). The insets show small, but significant changes (values represent the average of 5 different experiments +/− SD) at the level of the secondary structure of aSyn, in agreement the conformational changes observed in neuronal cells, where the distance between the N- and C-termini of aSyn is modified by DA treatment.

## Discussion

In PD, cell death affects primarily the dopaminergic neurons of the substantia nigra, but the nature of this selective vulnerability is still unclear. A common pathway, involving DA-dependent oxidative stress, has been put forward to explain the death of dopamine neurons. Defects in the sequestration of dopamine into synaptic vesicles in dopaminergic neurons from the substantia nigra, enabling undesired DA-aSyn interactions, may explain their increased vulnerability. It has been reported that DA can undergo auto-oxidation and form DA-quinone adducts with aSyn which prevent aSyn fibrillization and lead to the accumulation of toxic intermediates [5], [6], but the relevance of these findings in the context of living cells has been difficult to determine [32]. In the current study we sought to investigate whether DA influences the conformation of aSyn in primary neurons in culture.

We used FLIM to study alterations in the conformation of aSyn by monitoring the interactions between the N- and C-termini of the protein, as we had previously reported for studies in mammalian cell lines [31]. Here we demonstrate that aSyn adopts different conformations throughout the axon and dendrites. In vitro, purified aSyn does not display any secondary structure, and is considered a natively unfolded protein [10], [33], but it is highly likely that it adopts specific conformations inside neurons in order to perform its normal function(s). Our data show that different subcellular microenvironments, with potentially different redox conditions, lipid compositions, or other conditions known to influence the behavior of aSyn in vitro, afford aSyn the possibility of adopting distinct conformations inside living cells. For example, lipid rafts mediate the synaptic localization of aSyn in neurons [34], which may also explain the selective distribution of aSyn. Interestingly, we were not able to identify a specific association of a particular aSyn conformation with any subcellular organelle, suggesting local microenvironments may be more important in determining the structure/function of the protein. The interaction of aSyn with synaptic vesicles is highly dynamic [34], which may also explain the variety of aSyn conformations detected throughout the axons and dendrites.

Our data also demonstrate that aSyn changes its structure in response to DA, or possibly dopamine oxidation by-products, adopting a conformation where its N- and C-termini become closer together. DA or DA by-products inhibit aSyn fibril formation, which may, in turn, lead to the accumulation of aSyn oligomeric species via an alternative folding pathway [5], [6], [35], [36]. Although our results do not show whether the DA-induced change in conformation of aSyn is the precursor for the formation of toxic oligomeric species, our data support the model that DA-induced conformational changes in aSyn, either through a direct covalent interaction or indirectly, may favor changes in the oligomerization state of the protein which may explain the increased vulnerability of dopaminergic neurons in comparison to others. These conformational changes may underlie the recently reported effect of dopamine-modified aSyn on autophagy mediated degradation of the protein, and its subsequent impact on misfolded protein degradation in cells [16].

Defining the role of the identified aSyn conformations will shed light into the pathogenic mechanisms involved in PD, and may pave the way for the identification of novel targets for therapeutic intervention in different synucleinopathies.
